# Supplementary material for: Catalytic and Stoichiometric Baeyer–Villiger Oxidation Mediated by Nonheme Peroxo-Diiron(III), Acylperoxo, and Iodosylbenzene Iron(III) Intermediates
Source: Molecules. 2022 Apr 28;27(9):2814. doi: 10.3390/molecules27092814 (PMC9100052; doi:10.3390/molecules27092814)
Supplement: Supplementary file 1 [file molecules-27-02814-s001.zip › molecules-1683215-supplementary.pdf]

Supporting Information

Catalytic and Stoichiometric *Baeyer-Villiger* Oxidation Mediated by Nonheme Peroxo-Diiron(III), Acylperoxo and Iodosylbenzene Iron(III) Intermediates

Dóra Lakk-Bogáth [1], Miklós István Szávuly [1], Patrik Török [1] and József Kaizer [1,*]

[1] *Research Group of Bioinorganic and Biocoordination Chemistry, University of Pannonia, H-8201 Veszprém, Hungary*

* *Correspondence: kaizer@almos.uni-pannon.hu; Tel.: +36-88-62 4720.*

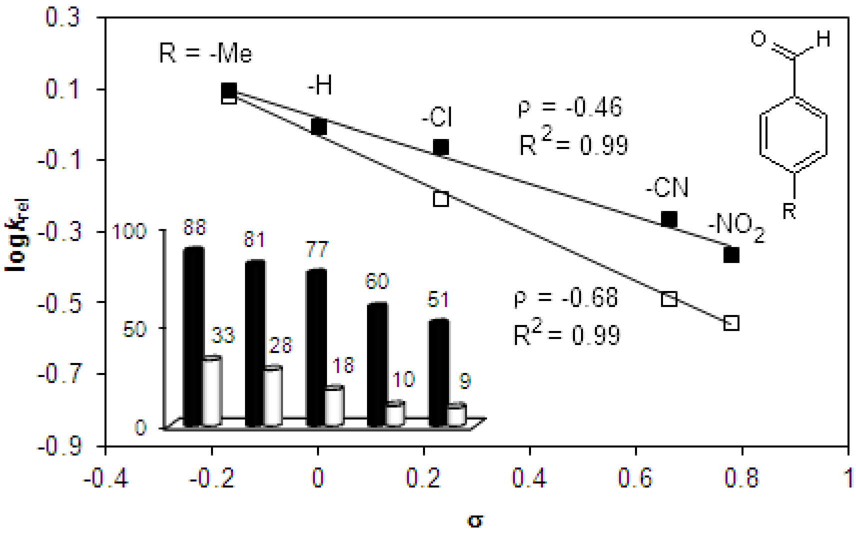

Figure S1. Hammett plot for the *para*-substituted benzaldehydes in the B.-V. reactions without **Fe(PBT)** (□), and with **Fe(PBT)** (■) in MeCN at 60 °C. Inset: yields in the reactions without **Fe(PBT)** (white), and with **Fe(PBT)** (black). $[\mathbf{Fe(PBT)}]_0 = 1.00 \times 10^{-5}$ M, $[\text{cyclohexanone}]_0 = 1.00 \times 10^{-2}$ M, $[\text{aldehyde}]_0 = 1.50 \times 10^{-1}$ M under O_2.

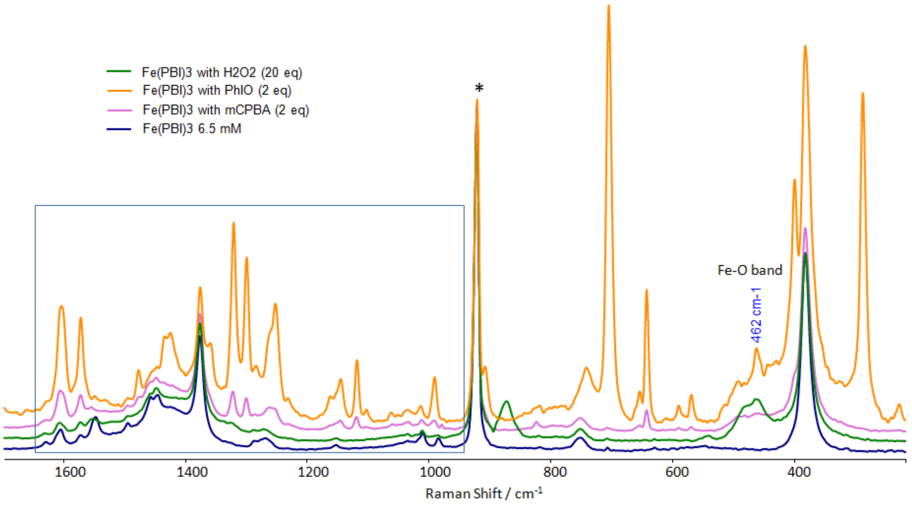

Figure S2. rRaman spectra of $\mathbf{3^{PhIO}}$, $\mathbf{3^{H2O2}}$ and $\mathbf{3^{mCPBA}}$ complexes.

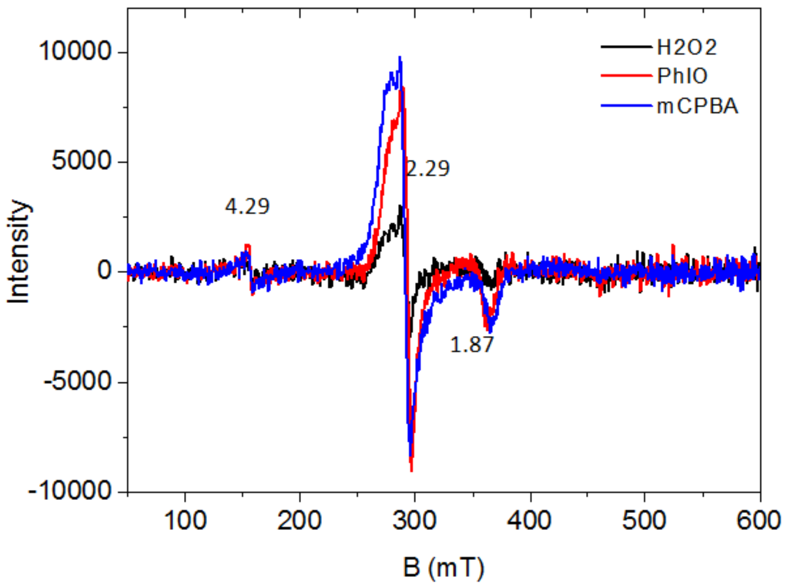

Figure S3. EPR spectra of $\mathbf{3^{PhIO}}$, $\mathbf{3^{H2O2}}$ and $\mathbf{3^{mCPBA}}$ complexes.
